# Supplementary material for: Traditional Knowledge of the Utilization of Edible Insects in Nagaland, North-East India
Source: Foods. 2020 Jun 30;9(7):852. doi: 10.3390/foods9070852 (PMC7404660; doi:10.3390/foods9070852)
Supplement: Supplementary file 1 [file foods-09-00852-s001.pdf]

<u>QUESTIONNAIRE FORMAT (by Lobeno Mozhui & L.N.Kakati)</u>

Name of the informant: ____

Age:

- ☐ **25-34**
- ☐ **35-44**
- ☐ **45-54**
- ☐ **55-64**
- ☐ **65-74**
- ☐ **75-84**
- ☐ **85-94**
- ☐ **95+**

Sex:

- ☐ **Male**
- ☐ **Female**

Community: ______________ Local dialect: ______________________

Qualification:

- ☐ **Below high school**
- ☐ **Above high school**

Occupation:

- ☐ **Government servant**
- ☐ **Farmer**
- ☐ **Unemployed**

Informant:

- ☐ **Key informant**
- ☐ **General informant**

Contact No.: _______________ Strength of family: ___________________

Place of interview:

a. Name of place/ village: ____

b. Location/ District: ____

c. Other details: ____

d. Signature: ____

1. Do you eat insects?

☐ **Yes**

☐ **No**

2. If yes, can you name the edible insects consumed by you?

**Sl. no**	**Common name**	**Local name**	**Seasonal availability**	**Edible stage**	**Mode of consumption**	**Therapeutic value**	**Myths/ Folklore**
1							
2							
3							
4							
5							

3. If no, can you name the edible insects consumed by others?

a. Local name of the insects: ______________________________

b. Name of the tribe consuming the insects: ____________________

c. Edible parts: ______________________________________

d. Edible stage: ______________________________________

e. Habitat of the insects: **Terrestrial/Aquatic/Burrowing**

f. Time of collection: __________________________________

g. Seasonal availability: ________________________________

h. Mode of collection: __________________________________

i. Mode of preparation and consumption: ___________________

4. Are there any indigenous methods for capturing insects?

☐ **Yes**

☐ **No**

5. If yes, can you name them and explain in details?

6. Are chemicals used for capturing insects?

☐ **Yes**

☐ **No**

7. If yes,

a. Name of the chemical: ________________________

b. Name of the insect captured: ___________________

8. Do you preserve the edible insects for future consumption?

☐ **Yes**

☐ **No**

9. If yes, then how are they preserved?

☐ **Fermented**

☐ **Smoked**

☐ **Sundried**

☐ **As Pickle**

Others: ____

10. Is entomophagy (insect consumption) popular in the region?

☐ **Yes**

☐ **No**

11. If yes, what are the most popular edible insects? Name them.

12. How often do you take/consume insects?

☐ **Everyday**

☐ **Once in a week**

☐ **Several days in a week**

☐ **Never**

13. Are insects reared?

☐ **Yes**

☐ **No**

14. If yes, name of the reared insects and technique used for domestication?

15. Are insects marketed?

☐ **Yes**

☐ **No**

16. If yes,

a. Name of the marketing place: ___________________________________

b. Name of the insects sold at the markets: ___________________________

c. Rate of the edible insects: ______________________________________

17. What are the insect and insect products sold by you? **(For insect farmers/sellers)**

a. Name of the insect/ insect product: _______________________________

b. Rate of selling: ____

c. Quantity of insects sold per day: _________________________________

d. Income on weekly basis (in rupees):

☐ **0- 5,000/-** ☐ **5,001-10,000/-** ☐ **ve 10,000/-**

18. Are insects used for cultural practices?

☐ **Yes**

☐ **No**

19. If yes, name of the insects used and how?

20. Are there any insect based folk stories in your community?

☐ **Yes**

☐ **No**

21. If yes, narrate the story.

22. Do you prefer insects over meat?

☐ **Yes**

☐ **No**

a. If yes, name of the insects preferred and why?

b. If no, why do you believe meat is better?

23. Are edible insects used in animal feed?

☐ **Yes**

☐ **No**

24. If yes,

a. Name of the insect used: ____________________

b. Mode of preparation: ____________________

25. Are edible insects used as baits?

☐ **Yes**

☐ **No**

26. If yes,

a. Name of the insect used: ____________________

b. Mode of use: ____________________

27. Do you manage insect pest for your cultivated crops?

☐ **Yes**

☐ **No**

28. If yes,

a. Chemical or non-chemical: ____________________

b. Regular or irregular use: ____________________

29. Are there any measures initiated to conserve edible insects?

☐ **Yes**

☐ **No**

30. If yes,

a. Name of the community/organization: ____________________

b. Methods used for conservation: ____________________

31. Give your feedback on availability of edible insects in the region.

- [ ] **Increased**
- [ ] **Decreased**

32. Does the younger generation practice entomophagy?

- [ ] **Yes**
- [ ] **No**

33. How important are insects according to you?

Date of collecting information ____________________________

Place of collecting information ___________________________

Name of enumerator ___________________________________
